# Draft Genome Sequence of a Novel Nicotine-Degrading Bacterium, *Pseudomonas plecoglossicida* TND35

**DOI:** 10.1128/genomeA.01162-14

**Published:** 2015-07-16

**Authors:** Gurusamy Raman, Natarajan Sakthivel, SeonJoo Park

**Affiliations:** a Department of Life Sciences, Yeungnam University, Gyeongsan, Gyeongbuk, Republic of Korea; b Department of Biotechnology, School of Life Sciences, Pondicherry University, Puducherry, India

## Abstract

*Pseudomonas plecoglossicida* TND35 is a potent nicotine-degrading bacterium. The draft genome sequence of strain TND35 contains 6,209,227 bp, 5,511 coding genes, and a G+C content of 62.3%. It encompasses genes related to catabolism of nicotine, *N*-heterocyclic aromatic compounds, heavy metal degradation, and butanol biosynthesis.

## GENOME ANNOUNCEMENT

Tobacco wastes that contain the principle alkaloid nicotine are harmful to human health and pollute the environment (1). These wastes are largely accumulated during the tobacco manufacturing process (2). Biological methods efficiently clean up toxic and hazardous wastes in the contaminated sites. A specific group of nicotine-degrading bacteria is involved in the degradation of nicotine (3). A novel nicotine-degrading bacterium, *Pseudomonas plecoglossicida* TND35, was isolated, identified, and deposited at the bacterial culture collection center, Department of Biotechnology, Pondicherry University, India. The 16S rRNA sequence of strain TND35 was deposited at GenBank database under accession no. JQ660543. This bacterium utilizes nicotine and employs a variant of the pyrrolidine pathway of nicotine biodegradation (1). However, the genome sequence of strain TND35 has not been sequenced yet. The genome sequence will provide insight into the molecular mechanism of its nicotine biodegradation.

The genome sequence of strain TND35 was determined by Illumina High-Seq 2000 (Macrogen, Inc., South Korea). A total of 31,406,636 reads were generated from a 100-bp paired-end library (total reads, 14,082,296; ~100-fold coverage) and 8-kb mate-pair libraries (total reads, 17,324,340; ~100-fold coverage). Sequence trimming and *de novo* assembly were performed using CLC-Genomics-Workbench, v7.0.4 (CLC-Bio, Denmark) and generated 69 contigs (>1000 bp) with an *N*_50_ length of 272,736 bp, a maximum contig size of 573,487, and an average size of 90,008 bp. These contigs were ordered using CONTIGuator v2.3 (4) with its closely related genome *Pseudomonas monteilii* SB3078 as a reference (GenBank accession no. CP006978.1). Forty-seven contigs contained in the 6.15-Mb genome were aligned with the reference genome. Sequences not mapped with the reference genome corresponding to 22 contigs, which are comprised of 58,201 nucleotides, were added later to the draft genome of strain TND35. These scaffolds were used for annotation using the RAST server (5) and the NCBI Prokaryotic Genomes and Automatic Annotation Pipeline, v2.7 (6). The scaffolds were searched against the KEGG database to analyze metabolic pathways and gene functions (7). Glimmer3 and GeneMarkS were used for the prediction of structural genes (8). RNAmmer and tRNAscan-SE were used to identify rRNAs and tRNAs (9, 10).

The draft genome sequence of strain TND35 contains 6,209,227 bp with a G+C content of 62.3%. It encodes 5,511 genes which include 5,397 predicted coding sequences (CDS), 49 pseudogenes, 60 tRNAs, 4 rRNAs, one non-coding RNA (ncRNA), and 36 frameshifted genes. The genome covers a total of 530 subsystems, which includes 4,106 CDS in total. The genome includes genes related to catabolism of *N*-heterocyclic aromatic compounds. The nicotine-degrading gene *hsp* was identified in the genome sequence of strain TND35. This key gene is very important in the conversion of 6-hydroxy-3-succinoyl pyridine to 2,5-dihydroxypyridine of the pyrrolidine pathway of *Pseudomonas* bacteria (11–13). Based on these findings, the strain TND35 follows the pyrrolidine pathway of nicotine degradation. The announcement of this genome information will allow further studies on the molecular mechanisms of nicotine-degrading genes and other genes related to *N*-heterocyclic aromatic compound degradation.

### Nucleotide sequence accession number.

This draft sequence has been deposited at DDBJ/EMBL/GenBank under accession no. JOJY00000000. The version described here is the first version.
